# A convenient and practical index for predicting the induction response in adult patients with hemophagocytic lymphohistiocytosis: ferritin/platelet ratio

**DOI:** 10.1007/s00277-023-05606-7

**Published:** 2024-01-10

**Authors:** Cuicui Feng, Zhengjie Hua, Lingbo He, Shuyan Yao, Heshan Zou, Yingxin Zhu, Zhao Wang, Yini Wang

**Affiliations:** 1 Department of Hematology, Capital Medical University Affiliated Beijing Friendship Hospital, Beijing, 100050 China; 2 Department of Hematology, Capital Medical University Affiliated Beijing Anzhen Hospital, Beijing, 100029 China; 3 Department of General Medicine, Capital Medical University Affiliated Beijing Friendship Hospital, Beijing, 100050 China

**Keywords:** Hemophagocytic lymphohistiocytosis, Induction therapy response, Serum ferritin, Platelets, Ferritin/platelet ratio

## Abstract

Hemophagocytic lymphohistiocytosis (HLH) is a hyperinflammatory syndrome with high mortality rate. The response to induction therapy is an important factor affecting survival. The purpose is to investigate laboratory predictors for induction response in adult patients with HLH, which are convenient, practical, and timeliness. Clinical data from January 2017 to December 2020 was retrospectively analyzed, and 269 patients were included. Patients were divided into remission and non-remission groups according to their induction response, 177 in the remission group, and 92 in the non-remission group. We reviewed general characteristics and analyzed the predictive value of serum ferritin, triglycerides, alanine aminotransferase (ALT), and blood cells before and 1–4 weeks after induction therapy for induction response by univariate analysis, ROC curves, etc. There was a correlation between serum ferritin, ALT, leukocytes, neutrophils, hemoglobin, platelets, and induction response (*P* < 0.05). Serum ferritin and platelets 1–4 weeks after induction therapy, respectively, might be a good predictor for induction response in adults with HLH, with *AUC* values close to or greater than 0.7. We established a new clinical model of the ferritin/platelet ratio. The results showed that the ferritin/platelet ratio at 1–4 weeks after induction therapy might be a practical index for predicting induction response, which significantly improved the area under the ROC curve (*AUC* > 0.75). Patients with a ferritin/platelet ratio > 16.08 at 2 weeks after induction therapy may have a relatively poor induction response. Ferritin/platelet ratio after induction therapy can be a good predictor for induction response in adult patients with HLH.

**Supplementary Information:**

The online version contains supplementary material available at 10.1007/s00277-023-05606-7.

## Introduction

Hemophagocytic lymphohistiocytosis (HLH) is an abnormally immune-activated disorder, a hyperinflammatory syndrome due to abnormal activation of macrophages, natural killer NK cells, and cytotoxic T cells, resulting in cytokine storms, phagocytic phenomena, multiorgan infiltration, and dysfunction [1]. The main clinical features are persistent fever, splenomegaly, hemophagocytosis in bone marrow, and cytopenia. In recent years, physicians’ awareness of HLH has gradually increased. According to incomplete statistics in 2019 [2], the annual incidence of HLH in China is about 1.04/1,000,000. As the largest tertiary referral center specializing in HLH in China, our center admits and treats about 250 patients per year, with about 100 first-time diagnosed patients. The mortality rate of HLH is high and fluctuates widely, ranging from 26.5 to 74.8% according to the etiology [3]. Therefore, early diagnosis and prompt treatment are essential. Studies have shown that up to 40% of HLH cases occur in adults. Response at 8 weeks after induction therapy in patients with HLH is one of the most relevant factors affecting overall survival, and it is a good prognostic factor [4]. Wang et al. found that the response to induction therapy was significantly related to survival [5]. Thus, unlike previous studies addressing overall survival, the response to induction therapy deserves our attention. Early identification of high-risk patients with poor induction response may help in the management of HLH and the prediction of prognosis. However, there are relatively few studies on predictors of induction response; therefore, the focus of this study was to analyze laboratory indicators that can help in the early prediction of response to induction therapy in adult HLH patients.

Currently, many studies focus on investigating new biological indicators, such as cytokines, sCD25, and NK cell activity, to predict the prognosis of HLH. Despite their high sensitivity, they are not available in all medical institutions. The timeliness and practicability of laboratory indicators for predicting the efficacy of induction therapy for HLH is equally important. Therefore, we focused on traditional laboratory indicators that have the advantages of being easily accessible, easy to monitor, and time-sensitive. Taking the current internationally recognized therapeutic evaluation indexes of HLH as the main breakthrough point, including sCD25, serum ferritin, triglycerides, alanine aminotransferase (ALT), blood cells, phagocytic phenomena, and consciousness, we analyzed the traditional laboratory parameters such as serum ferritin, triglycerides, alanine aminotransferase (ALT), and blood cells and investigated their relationship to induction response and their predictive value in HLH patients. It is hoped that monitoring these indicators can help clinicians make early assessments and judgments about the response to induction therapy.

## Methods

### Patients

**Fig. 1:**
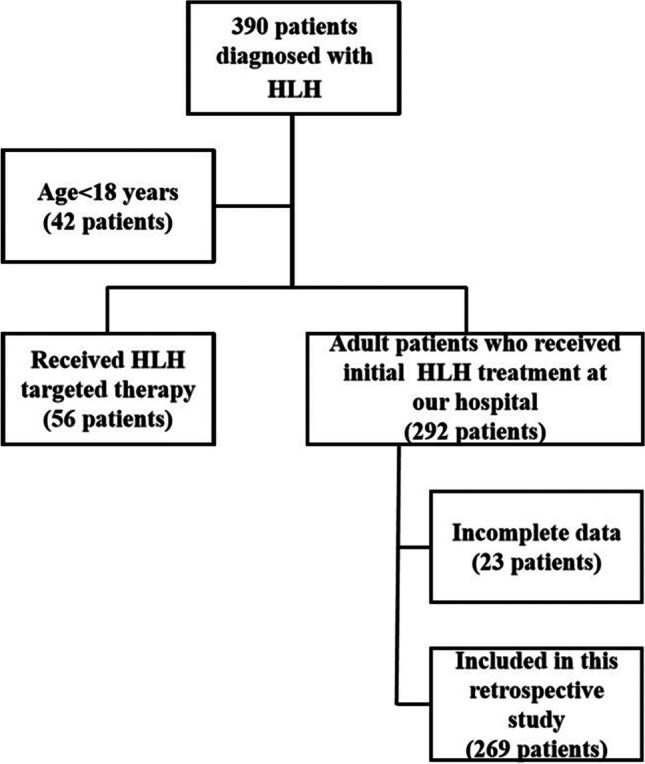
Diagram of enrolled patients

This research was in line with the Declaration of Helsinki and approved by the Ethics Committee at Beijing Friendship Hospital, Capital Medical University, and obtained written informed consent. A total of 390 patients admitted with a preliminary diagnosis of HLH between January 2017 and December 2020 were retrospectively analyzed. Inclusion criteria: (1) Met the HLH-2004 diagnostic criteria [6]; (2) age ≥ 18 years; and (3) patients who received initial HLH treatment at our hospital. Exclusion criteria: (1) age < 18 years; (2) receiving HLH-targeted therapy; and (3) incomplete clinical data. We ultimately analyzed 269 adult HLH patients who received initial induction therapy at our institution (Fig. 1), with a median age at diagnosis of 37 years, the youngest being 18 years and the oldest being 81 years. HLH was classified according to etiology into infectious diseases (*n* = 129), malignant tumors (*n* = 82), autoimmune diseases (*n* = 33), primary HLH (*n* = 5), and idiopathic HLH (HLH of unknown etiology) (*n* = 20). Patients were divided into the remission group (*n* = 177) and the non-remission group (*n* = 92) based on their response to induction treatment.

### Parameters associated with HLH

General information about the patient diagnosed with HLH (including age and gender), as well as laboratory results at the time of the patient’s initial admission, including HLH-2004 diagnostic indicators (fever, splenomegaly, hemophagocytosis in bone marrow, cytopenias, triglycerides, fibrinogen, natural killer (NK) cytotoxic activity, sCD25, and serum ferritin), etiology, baseline biochemical parameters, and induction treatment regimens. Besides, we collected values of serum ferritin, triglycerides, alanine aminotransferase (ALT), and blood cells at 1, 2, 3, and 4 weeks after induction therapy.

### Treatments

All patients included in this study received a different induction therapy according to their condition, including HLH-1994/2004 regimen (including etoposide, dexamethasone, cyclosporine, intrathecal methotrexate, and dexamethasone, *n* = 119), DEP regimen (including doxorubicin liposome, etoposide, and methylprednisolone, *n* = 87) [7], L-DEP regimen (pegaspargase or asparaginase, doxorubicin liposome, etoposide, and methylprednisolone, *n* = 63).

### Response assessment

The induction response evaluation criteria was mainly based on the efficacy evaluation criteria proposed by Marsh et al. [8], which was recognized in the international consensus and revised by Wang et al. Patients were divided into the remission group (complete remission CR and partial remission PR) and the non-remission group (ineffective NR after treatment) according to induction response. The response assessment refers to the evaluation of the induction response according to the patient’s symptoms and laboratory indicators at an interval of 1 week after induction treatment, with failure to remit during induction therapy as the outcome event, which is classified as the non-remission group; the patients with continuous remission within 8 weeks of the induction treatment are classified as the remission group.

### Statistical analysis

All data were processed by SPSS 26.0 statistical software. Measurement data conforming to normal distribution were expressed as *mean* ± standard deviation, and those not conforming to normal distribution were expressed as median and percentile. Independent sample *t*-test, Mann-Whitney *U* test, and chi-square test were employed to make a comparison between groups for normally distributed variables, non-normally distributed variables, and categorical variables, respectively. Multivariate analysis was conducted using logistic regression. The ROC curves were applied to assess the predictive value of each indicator and the optimal cut-off value. The Kaplan-Meier method was used to plot the curve, and Cox regression analysis was used to investigate the induction-treatment response of different stratification of the index. MedCalc statistical software was applied to compare the differences in *AUC* values of the indicators in each group. Two-tailed *P* < 0.05 was considered statistically significant.

## Results

### Patients’ characteristics

The basic condition of the patients at the time of admission and the characteristics of the laboratory indexes are shown in Supplementary Table 1. The results showed that all patients had febrile symptoms, 213 cases of splenomegaly (remission/non-remission group, 144/69), and 197 cases of hemophagocytosis in bone marrow (remission/non-remission group, 135/62). Before treatment, the median value of serum ferritin was 3652 μg/L (range, 84.16–75,000 μg/L); triglyceride was 2.38 mmol/L (range, 0.60–9.65 mmol/L), and ALT was 76 U/L (range, 3.13–2145 U/L). The median value of leukocytes was 2.71 × 10^9^/L (range, 0.01–32.85 × 10^9^/L); neutrophil was 1.59 × 10^9^/L (range, 0.00–25.9 × 10^9^/L); and hemoglobin was 90 g/L (range, 37–151 g/L). The median platelet value was 69 × 10^9^/L (range, 2–479 × 10^9^/L). The response rate for the HLH-1994/2004 regimen was 58% (69/119 patients); for the DEP regimen, it was 72% (63/87 patients), and for the L-DEP regimen, it was 71% (45/63 patients) (Supplementary Table 1).

Univariate analysis showed statistical differences in fibrinogen, leukocytes, hemoglobin, platelets, albumin, total bilirubin, direct bilirubin, indirect bilirubin, HDL, urea, calcium ions, sodium ions, glucose, and etiology between the remission and non-remission groups. Among them, fibrinogen, leukocytes, hemoglobin, platelets, albumin, HDL, calcium ions, and sodium ions were significantly lower in the non-remission group than in the remission group (*P* < 0.05), and total bilirubin, direct bilirubin, indirect bilirubin, urea, and blood glucose were significantly higher in the non-remission group than in the remission group (*P* < 0.05). Other indicators collected were not statistically significant between the two groups (*P* > 0.05) (Supplementary Table 1). To exclude the interaction between the factors, we conducted a multifactorial analysis of these statistically different indicators above. The results showed that there was no significant difference (*P* > 0.05) between the remission and non-remission groups for any of the above indicators (Supplementary Table 2).

### Relationship among induction response with serum ferritin

Statistical analysis of serum ferritin data before and after induction therapy in the remission and non-remission groups was performed by independent sample non-parametric test. The results showed that there was a significant difference in serum ferritin values 1–4 weeks after induction therapy between the two groups, and serum ferritin values in the non-remission group were significantly higher than those in the remission group (*P* < 0. 05) (Supplementary Table 3). The difference in serum ferritin before induction therapy between the two groups was not statistically significant (*P* > 0.05) (Supplementary Table 1).

**Fig. 2:**
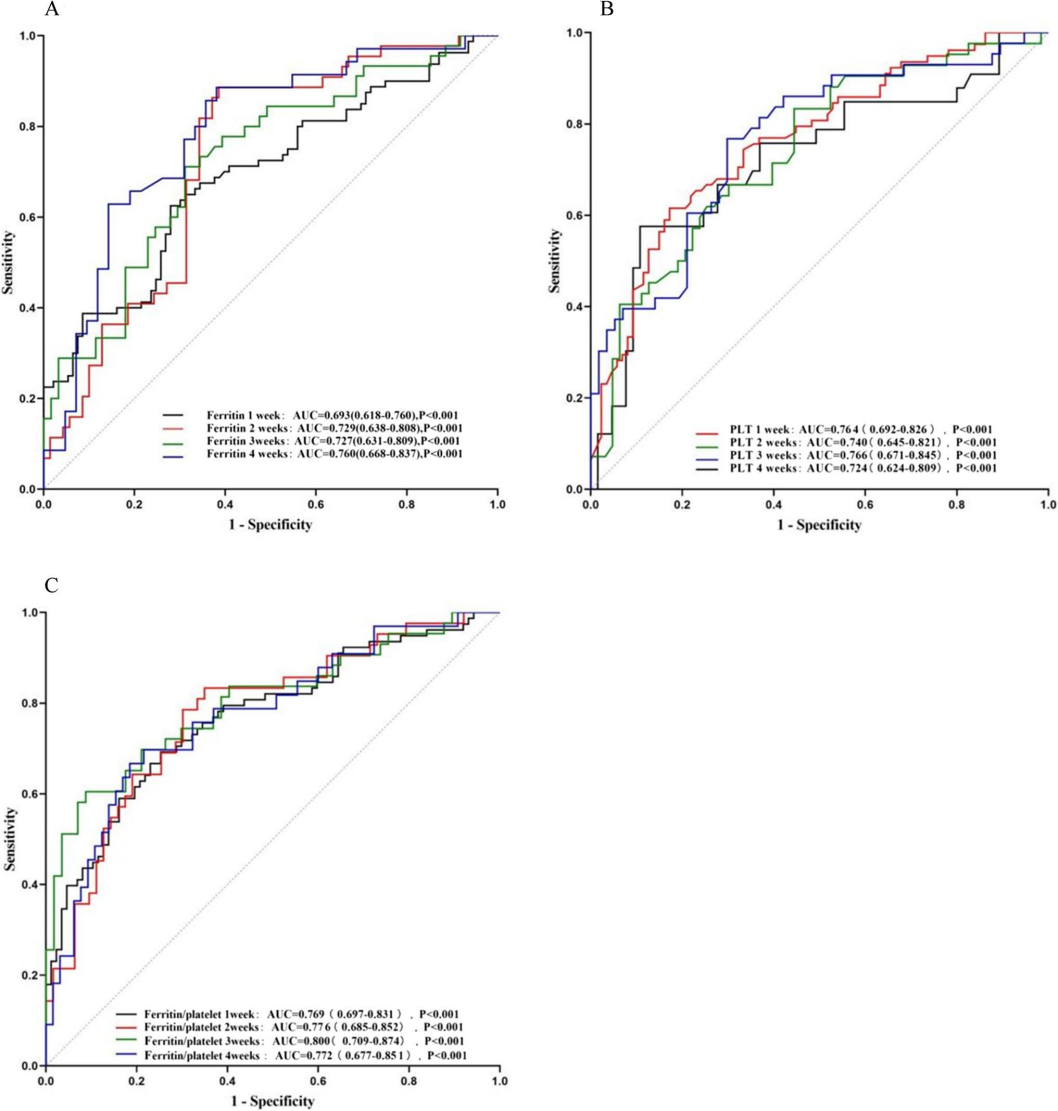
**A** ROC analysis of serum ferritin 1–4 weeks after treatment in predicting induction response. *AUC*s were all greater than 0.5. Serum ferritin 4 weeks after induction therapy had the largest area under the curve for predicting the response to induction therapy. The *AUC* of serum ferritin 2 weeks after induction therapy was the second. **B** ROC analysis of platelets in predicting induction response. *AUC*s were all greater than 0.5. The area under the ROC curve for platelets 3 weeks after induction therapy was the largest. **C** ROC analysis of ferritin/platelet ratio in predicting induction response. *AUC*s were all greater than 0.75. The *AUC* of ferritin/platelet ratio 3 weeks after induction therapy was the largest. The *AUC* of ferritin/platelet ratio 2 weeks after induction therapy was the second

The ROC curve was further applied to analyze the value of serum ferritin levels 1-4 weeks after induction therapy in predicting the response to HLH induction therapy, and the results showed that the area under the ROC curve was greater than 0.5. Serum ferritin 4 weeks after induction therapy had the largest area under the curve for predicting the response to induction therapy (*AUC* = 0.760, 95% *CI* 0.668–0.837, *P* < 0.001); serum ferritin 2 weeks after induction therapy had the next highest *AUC* value (*AUC* = 0.729, 95% *CI* 0.638–0.808, *P* < 0.001), with a sensitivity of 88.60%, a specificity of 61.40%, and an optimal threshold of 1256 μg/L; and the areas under the ROC curves at 1 and 3 weeks after induction therapy were 0.693 and 0.727, respectively (*AUC* = 0.693, 95% *CI* 0.618–0.760, *P* <0.001; *AUC* = 0.727, 95% *CI* 0.631–0.809, *P* < 0.001) (Supplementary Table 4; Fig. 2).

### Relationship among induction response with serum ferritin decline ratio

In order to exclude the influence of individual baseline serum ferritin levels on the results, we analyzed the rate of serum ferritin decline before and after treatment, Serum ferritin decline ratio = (pre-treatment serum ferritin − post-treatment serum ferritin) / pre-treatment serum ferritin. Statistical analysis by ROC curves showed that the serum ferritin decline ratios at 1, 3, and 4 weeks after induction therapy were statistically different between the remission and non-remission groups, and the serum ferritin decline ratio at 4 weeks after induction therapy had a relatively large area under the curve (*AUC* = 0.675, 95% *CI* 0.562–0.789, *P* = 0.003) for predicting the response to induction therapy, with a sensitivity of 80.6%, specificity of 60%, and the best cut-off value 44.13% (Supplementary Table 5, Supplementary Figure 1).

### Relationship among induction response with triglycerides and ALT

Triglycerides and ALT 1–4 weeks after induction therapy between the remission group and the non-remission group were analyzed by independent sample non-parametric test, and the results showed that triglycerides were not statistically different between the two groups (*P* > 0.05); ALT 1, 2, and 3 weeks after induction therapy were not statistically different between the two groups (*P* > 0.05), and ALT 4 weeks after induction therapy was significantly higher in the non-remission group than that in the remission group (*P* < 0.05) (Supplementary Table 3). The results of the ROC curve analysis showed that the *AUC* of ALT at 4 weeks after induction therapy for predicting the induction response was greater than 0.5 (*AUC* = 0.669, 95% *CI* 0.553–0.785 *P* = 0.007), with a sensitivity of 75.80%, specificity of 57.40%, and the optimal cut-off value of 38.5 U/L (Supplementary Table 4, Supplementary Figure 2).

### Relationship among induction response with blood cells

The differences in blood cells between the two groups at 1–4 weeks after induction therapy were analyzed by independent samples non-parametric test, and the results showed that platelets 1–4 weeks after induction therapy were statistically different between the two groups, with the remission group being significantly higher than the non-remission group (*P* < 0.05); hemoglobin was significantly higher in the remission than in the non-remission group at 1, 2, and 4 weeks after induction therapy; and leukocyte and neutrophils values were significantly higher in the remission group than in the non-remission group at 1 and 2 weeks after induction therapy, and the difference was statistically significant (*P* < 0.05) (Supplementary Table 3).

ROC curves were analyzed for the statistically different indicators between the two groups to further explore their relationship with the response to induction therapy and their prognostic value. The results showed that the area under the ROC curve of leukocytes and neutrophils at 1–2 weeks after induction therapy was greater than 0.5. The *AUC* of hemoglobin at 1, 2, and 4 weeks after induction therapy were all greater than 0.5, of which the ROC of hemoglobin at 2 weeks after induction therapy had the largest area under the curve (*AUC* = 0.742, 95% *CI* 0.647–0.822, *P* < 0.001). Platelets at 1 to 4 weeks after induction therapy had a relatively large area under the curve, with an *AUC* of more than 0.7, and the highest *AUC* value was found 3 weeks after induction therapy (*AUC* = 0.766, 95% *CI* 0.671–0.845, *P* < 0.001) (Supplementary Table 4; Fig. 2).

### Relationship among induction response with ferritin/platelet ratio

The previous analysis showed that the *AUC* values of serum ferritin and platelets after induction therapy for predicting induction response were close to or greater than 0.7, respectively, suggesting a possible good predictive value. Elevated serum ferritin and reduced platelets were seen in the non-remission group compared with the remission group, so we ratioed the two to establish a clinical model of ferritin/platelet. Univariate analysis showed a significant difference in ferritin/platelet ratio 1–4 weeks after induction therapy between the remission and non-remission groups (Supplementary Table 3). The results of ROC curve analysis showed that ferritin/platelets 1–4 weeks after induction therapy had a good area under the curve for predicting induction response, with an *AUC* > 0.75 (Supplementary Table 4; Fig. 2).

The previous analysis showed that serum ferritin, platelets, and ferritin/platelet ratios after induction therapy might be statistically significant for predicting induction response. In addition, we applied MedCalc statistical software to analyze the differences between the three indexes in a horizontal comparison, and the *AUC* of the ferritin/platelet ratio was significantly higher than that of serum ferritin only at 1 and 3 weeks after induction therapy (Supplementary Table 6).

**Fig. 3:**
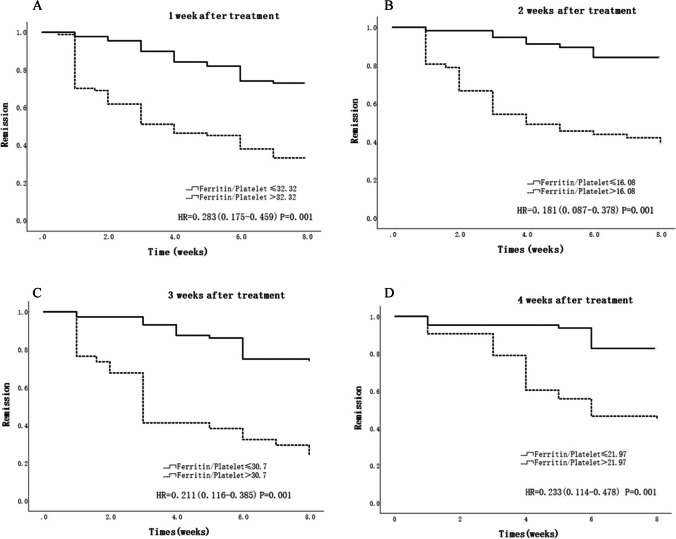
Response to induction therapy in patients with different ferritin/platelet ratio stratification 1–4 weeks after induction therapy was significantly different. 1 week: *HR* = 0.283 (0.175–0.459) *P* = 0.001; 2 weeks: *HR* = 0.181 (0.087–0.378) *P* = 0.001; 3 weeks: *HR* = 0.211 (0.116–0.385) *P* = 0.001; 4 weeks: *HR* = 0.233 (0.114–0.478) *P* = 0.001

Kaplan-Meier curves and Cox risk regression models were used to analyze differences in induction response in patients stratified by different ferritin/platelet ratios. The results showed that there was a statistically significant difference in induction response in patients stratified by different ferritin/platelet ratios from 1 week to 4 weeks after induction therapy (Fig. 3).

## Discussion

In this study, we combined serum ferritin and platelet to develop a clinical model of the ferritin/platelet ratio, which could help in the early prediction of adult HLH patients with poor induction response.

We first analyzed the relationship between patients’ general condition and laboratory indicators before treatment and induction response. Statistical differences were found in fibrinogen, leukocytes, hemoglobin, platelets, albumin, bilirubin, HDL, urea, calcium ions, sodium ions, blood glucose, and etiology between those in the two groups, which was supported by the results of Fardet et al. [9, 10]. However, a multifactorial analysis of the above indicators showed no independent risk factors. Considering the interrelatedness of laboratory indicators and the complex course of HLH, it seems inadequate to use only one static index before treatment to predict the induction response. Therefore, laboratory indicators after induction therapy equally deserve our attention and further study.

Serum ferritin is one of the most relevant reactants in the acute phase of HLH. Ferritin is mainly stored in macrophages [1], and extensive activation of macrophages may be responsible for the significant elevation of serum ferritin. Serum ferritin may also play a pro-inflammatory role [11]. Previous studies showed that serum ferritin was a useful indicator for diagnosis and predicting the disease activity and prognosis of HLH [12–14]. However, serum ferritin, as an acute-phase protein, has better value in reflecting the acute phase of disease; moreover, the complexity of HLH process makes serum ferritin levels susceptible to interference by various factors, such as infection. Therefore, we focused on the clinical value of predicting induction response. Monitoring serum ferritin at admission and 4 weeks after induction therapy continuously, we found that pre-induction serum ferritin had no significant correlation with induction response; the result was supported by Zhou et al [9, 12, 15]. Serum ferritin 1–4 weeks after induction therapy was significantly higher in the non-remission group, with *AUC* values close to or greater than 0.7. Therefore, we tended to believe that serum ferritin 1–4 weeks after induction therapy might be a good predictor for the induction response. Considering the differences in baseline serum ferritin among patients, we analyzed whether changes in serum ferritin could predict induction response. The results showed that a serum ferritin decline ratio at 4 weeks after induction therapy may be a good predictor, with an optimal threshold of 45%. Rand and Lin et al. proposed its clinical value of predicting survival in HLH as well [14, 16].

Triglyceride is an important indicator in the diagnosis and efficacy assessment of HLH. Zhou et al. found that triglycerides were an independent risk factor for overall survival in HLH [17]. Our results showed no statistical correlation between induction response and triglycerides. Elevated transaminases have been included as an important indicator for diagnosis of HLH in Hscore and the International Consensus [6, 18]. ALT is a significant indicator recommended in guidelines for the assessment efficacy of HLH [19]. Zhou et al. suggested that high ALT levels had a predictive value for poor prognosis of HLH [20]. However, there are still relatively few relevant studies. In this study, we found that ALT 4 weeks after induction therapy might predict induction response, and ALT greater than 38.5 U/L might indicate a poor response to induction therapy.

Decreased blood cells are one of the most important indicators for the diagnosis and outcome evaluation of HLH. Inflammatory cytokine storm produced in HLH leads to reduced white blood cells, neutrophils, platelets, and hemoglobin. Thrombocytopenia has been confirmed as a prognostic and long-term survival indicator of HLH in extensive studies [4, 10, 21]. We found that platelets 1–4 weeks after induction therapy may serve as a good predictor for induction response, with *AUC* > 0.7. In addition, there was a correlation between leukocytes, neutrophils, hemoglobin, and induction response at some time points after induction therapy, which might be related to the significant elevation of cytokines, acting on macrophages to cause endocytosis in HLH, supported by the studies of Bin and Pan et al. [22–24]. As important indexes for the assessment of HLH efficacy, they may have some value in predicting the induction response; however, the *AUC* value is about 0.6.

Based on the above studies, considering the complex course of HLH and its susceptibility to multiple factors such as severe infections, multiple organ failure, and HLH progression, the correlation and association between the indicators may help to complement each other and jointly reflect the response to induction therapy in HLH from different aspects, thus improving the accuracy of response prediction. By analyzing the above indicators, we found that each of the serum ferritin and platelets after induction therapy may be a good predictor of the response to induction therapy, respectively, with *AUC* values close to or greater than 0.7. Elevated serum ferritin and reduced platelets were seen in the non-remission group compared to the remission group, so we combined the two in a clinical model to analyze the relationship between the ferritin/platelet ratio and the response to induction therapy and its predictive value. We found that the ferritin/platelet ratio 1–4 weeks after induction therapy was statistically different between the two groups, and ROC analysis confirmed the predictive value of the ratio. The ratio increased the area under the ROC curve compared with each of the serum ferritin and platelets individually (*AUC* > 0.75), suggesting that it may have a better predictive significance. Among them, the ferritin/platelet ratio at 2 weeks after induction therapy for predicting induction response had a large area under the ROC curve, with *AUC* = 0.776, a sensitivity of 78.60%, and a specificity of 69.80%, which was more sensitive compared with that at 3 weeks after induction therapy (*AUC*_max_ = 0.800), and it helped to identify the high-risk patients with a poor induction response. On the other hand, it was favorable for early prediction and improved timeliness. This was in line with the timing of salvage therapy (i.e., salvage therapy can be initiated if there is no good response after 2–3 weeks of initial treatment for HLH) agreed by most scholars in the international arena. Our findings further support the importance of monitoring the ferritin/platelet ratio 2 weeks after induction therapy in adult HLH patients, and patients with a ratio > 16.08 may have a relatively poor induction response, which can help to prompt clinicians to initiate treatment change early.

This study innovatively proposed the ferritin/platelet ratio for HLH-induction response prediction. By intergroup comparison of the *AUC*s of serum ferritin, platelet, and ferritin/platelet, although the *AUC* of the ferritin/platelet ratio was significantly higher than that of serum ferritin only at 1 and 3 weeks after induction therapy, our results still suggested that all three indexes might have a better prognostic value for the response to induction therapy. Our conclusions can be further analyzed and validated in multicenter prospective clinical studies with larger sample sizes.

## Conclusion

Each of serum ferritin and platelets 1–4 weeks after induction therapy may be a good laboratory indicator for predicting the response to induction therapy in adults with HLH, respectively, and has the advantages of convenience, easy follow-up monitoring, and timeliness. The ferritin/platelet ratio at 1–4 weeks after induction therapy can be used for early prediction of induction response with good judgment value, and patients with ferritin/platelet ratio > 16.08 at 2 weeks after induction therapy may have a relatively poor response to induction therapy, which can help clinicians identify high-risk patients with poor response to induction at an early stage and adjust the treatment strategy in a timely manner.

### Supplementary information

ESM 1 (DOCX 73 kb)

## Data Availability

The datasets generated during and/or analyzed during the current study are available from the corresponding author on reasonable request.
