# Supplementary material for: A convenient and practical index for predicting the induction response in adult patients with hemophagocytic lymphohistiocytosis: ferritin/platelet ratio
Source: Ann Hematol. 2024 Jan 10;103(3):715–23. doi: 10.1007/s00277-023-05606-7 (PMC10867095; doi:10.1007/s00277-023-05606-7)
Supplement: Supplementary file 1 — (DOCX 73 kb) [file 277_2023_5606_MOESM1_ESM.docx]

Supplementary Table 1. General information and laboratory indices of the patients at the time of admission.

|  | Remission | Non-remission | P-value |
| --- | --- | --- | --- |
| Median age (years) | 37（27-51） | 36（28-52） | 0.497 |
| Gender (male/female) | 177(94/83) | 92(54/38) | 0.067 |
| Etiology | 177 | 92 | 0.019 |
| Infectious diseases | 82 | 47 |  |
| Malignancies | 52 | 30 |  |
| Autoimmune disorders | 25 | 8 |  |
| Primary HLH | 5 | 0 |  |
| No identified | 13 | 7 |  |
| Fever (All patients) | 177 | 92 |  |
| Splenomegaly(N/Y) | 177(33/144) | 92(23/69) | 0.940 |
| Hemophagocytosis  (N/Y) | 177(42/135) | 92(30/62) | 0.239 |
| TG (mmol/L) | 2.30(1.63-3.24) | 2.41(1.72-3.59) | 0.256 |
| FIB (g/L) | 1.95（1.33-2.96） | 1.64（1.07-2.41） | 0.035 |
| NK % activity | 15.08%（13.40%-16.55%） | 14.28%（13.10%-16.81%） | 0.456 |
| sCD25(pg/ml) | 19841(10076-34947) | 26403(14762-39523) | 0.096 |
| Admission ferritin (μg/L) | 3367（1283-8237） | 3870（1389-16500） | 0.090 |
| WBC (×10^9^/L) | 3.05（1.58-6.29） | 2.47（1.02-4.95） | 0.046 |
| NEUT (×10^9^/L) | 1.73（0.95-4.255） | 1.51（0.61-3.25） | 0.077 |
| HB (g/L) | 95（74-111） | 86（72-106） | 0.012 |
| PLT (×10^9^/L) | 77（38-165） | 54（21-105） | 0.001 |
| ALT (U/L) | 69.5(29-145.5) | 82(41-142) | 0.401 |
| AST (U/L) | 63.6(30.9-139.6) | 78(35-188) | 0.226 |
| AST/ALT | 1.005(0.63-1.64) | 1.088(0.66-1.81) | 0.600 |
| GGT(U/L) | 128.5(60-226.75) | 145(61-313) | 0.281 |
| ALP (U/L) | 164(94.25-285.5) | 186(96-353) | 0.112 |
| ALB (g/L) | 30.03±5.36 | 27.90±4.88 | 0.001 |
| TBIL(μmol/L) | 18.43(11.61-34.97) | 25.8(14.67-60.73) | 0.001 |
| DBIL (umol/L) | 5.60(2.92-123) | 9.29(4.06-32.67) | 0.001 |
| IBIL(μmol/L) | 12.95(8.5-23.67) | 16.31(10.23-34.20) | 0.019 |
| HDL (mmol/L) | 0.66（0.41-1.01） | 0.56（0.33-0.89） | 0.019 |
| LDL (mmol/L) | 2.24（1.71-2.92） | 2.02（1.43-2.68） | 0.121 |
| LDH (U/L) | 477（310.5-885） | 530（336.25-983.25） | 0.256 |
| Urea (mmol/L) | 4.81(3.55-7.08） | 5.71（3.89-7.37） | 0.001 |
| Cr(μmol/L) | 54.5（43.28-65.13） | 56.6（42.58-71.7） | 0.165 |
| Ca2+(mmol/L) | 2.05（1.95-2.17） | 2.00（1.88-2.12） | 0.002 |
| UA (umol/L) | 215.1（162.0-316.9） | 210.8（163.1-280.5） | 0.912 |
| BG(mmol/L) | 5.27(4.64-6.26) | 5.74(4.57-7.08) | 0.027 |
| Na2+ (mmol/L) | 136.25（133.95-138.43） | 135.7（131.38-138.2） | 0.037 |
| Induction treatment | 177 | 92 | 0.055 |

A two-tailed P < 0.05 was considered statistically significant. TG, triglyceride; FIB, fibrinogen; WBC, [white](javascript:;) [blood](javascript:;) [cell](javascript:;); NEUT, [neutrophile](javascript:;) [granulocyte](javascript:;); HB, hemoglobin; PLT, platelets; ALT, alanine aminotransferase; AST, aspartate aminotransferase; LDH, lactate dehydrogenase; TBIL, total bilirubin; DBIL, [direct](javascript:;) [bilirubin](javascript:;); IBIL, indirect bilirubin; HDL, [high-density](javascript:;) [lipoprotein](javascript:;); LDL, [low](javascript:;) [density](javascript:;) [lipoprotein](javascript:;); ALB, albumin; ALP, [alkaline](javascript:;) [phosphatase](javascript:;); UA, [uric](javascript:;) [acid](javascript:;); Cr, creatinine; Ca2+, calcium; BG, bloodglucose; Na2+, Natrium; ALB, albumin.

Supplementary Table 2. Multivariate analyses of factors that were statistically different between the two groups.

| Variables | Multivariate analysis | | |
| --- | --- | --- | --- |
|  | OR | 95%CI | P-value |
| FIB | 0.885 | 0.715-1.095 | 0.261 |
| WBC | 0.944 | 0.862-1.033 | 0.211 |
| HB | 0.997 | 0.983-1.010 | 0.628 |
| PLT | 1.000 | 0.996-1.004 | 0.866 |
| ALB | 0.966 | 0.891-1.047 | 0.401 |
| TBIL | 1.005 | 0.994-1.016 | 0.335 |
| DBIL | 1.000 | 0.986-1.015 | 0.957 |
| IBIL | 0.990 | 0.974-1.006 | 0.215 |
| HDL | 1.611 | 0.804-3.226 | 0.179 |
| Urea | 1.075 | 0.997-1.160 | 0.061 |
| Ca2+ | 0.785 | 0.082-7.500 | 0.833 |
| BG | 1.132 | 1.000-1.282 | 0.050 |
| Na2+ | 0.935 | 0.874-1.002 | 0.056 |
| Etiology |  |  | 0.510 |

A two-tailed P < 0.05 was considered statistically significant. FIB, fibrinogen; WBC, [white](javascript:;) [blood](javascript:;) [cell](javascript:;); HB, hemoglobin; PLT, platelets; TBIL, total bilirubin; DBIL, [direct](javascript:;) [bilirubin](javascript:;); IBIL, indirect bilirubin; HDL, [high-density](javascript:;) [lipoprotein](javascript:;); Ca2+, calcium; BG, bloodglucose; Na2+, Natrium; ALB, albumin.

Supplementary Table 3. Correlation analysis of indicators and response to induction therapy.

| Indicators | Times after treatment(weeks) | Remission | Non-remission | P-value |
| --- | --- | --- | --- | --- |
| Ferritin  (ug/L) | 1 | 1623（930-3189） | 3062（1440-10661） | ＜0.001 |
|  | 2 | 924（450-1923） | 1765（1422-3731） | ＜0.001 |
|  | 3 | 1150（609.5-2175.5） | 2374.5（1478.250-6947.750） | ＜0.001 |
|  | 4 | 809（345.5-1442） | 2595（1165-4394） | ＜0.001 |
| TG (mmol/L) | 1 | 2.1（1.53-2.90） | 2.31（1.56-3.27） | 0.283 |
|  | 2 | 1.73（1.04-2.715） | 2.095（1.07-3.205） | 0.298 |
|  | 3 | 2.03（1.16-2.975） | 2.23（1.185-4.2025） | 0.178 |
|  | 4 | 1.68（1.075-2.725） | 2.16（1.33-3.21） | 0.138 |
| ALT  (U/L) | 1 | 53（25.25-107.25） | 54（29-127） | 0.545 |
|  | 2 | 36（23-65） | 37（21.5-97.75） | 0.348 |
|  | 3 | 38（21-62.5） | 49.5（20-133.75） | 0.087 |
|  | 4 | 33（16.5-57.5） | 52（32-120） | 0.007 |
| WBC  (×10^9^/L) | 1 | 3.745（2.0600-5.5300） | 1.84（0.55-4.03） | ＜0.001 |
|  | 2 | 3.905（2.19-5.82） | 2.305（1.30-6.23） | 0.036 |
|  | 3 | 3.29（2.46-7.695） | 3.36（1.965-6.495） | 0.524 |
|  | 4 | 4.55（3.38-7.045） | 4.22（1.7-6.94） | 0.191 |
| NEUT (×10^9^/L) | 1 | 2.85（1.67-4.1825） | 1.21（0.26-3.6） | 0.001 |
|  | 2 | 2.35（1.41-4.41） | 1.55（0.64-3.95） | 0.044 |
|  | 3 | 2.46（1.565-5.74） | 2.655（1.3075-5.12） | 0.631 |
|  | 4 | 3.20（2.32-5.79） | 2.77（1.18-5.82） | 0.355 |
| HB  (g/L) | 1 | 89（73-102） | 76（68-88） | 0.002 |
|  | 2 | 92（82-112） | 77（68-89） | ＜0.001 |
|  | 3 | 89（83-107） | 88（75-105） | 0.142 |
|  | 4 | 103（81-115） | 82（67-96） | 0.004 |
| PLT  (×10^9^/L) | 1 | 129（75-221） | 38（14-94） | ＜0.001 |
|  | 2 | 130（74-217） | 70（22-124） | ＜0.001 |
|  | 3 | 175（110-219） | 101（26-127） | ＜0.001 |
|  | 4 | 171（102-229） | 69（41-145） | ＜0.001 |
| Ferritin/platelet Ratio | 1 | 14.33(5.82-32.94) | 98.56(24.29-753.58) | ＜0.001 |
|  | 2 | 7.50(3.60-20.05) | 33.88(16.58-91.41) | ＜0.001 |
|  | 3 | 7.55(3.31-14.53) | 51.20(10.44-167.84) | ＜0.001 |
|  | 4 | 4.74(1.25-17.61) | 37.62(9.26-123.66) | ＜0.001 |

Supplementary Table 4. ROC analysis of indicators in predicting induction response.

| Indicators | Times after treatment  (weeks) | AUC | 95%CI | P-value | Sensitivity | Specificity | Cut-off value |
| --- | --- | --- | --- | --- | --- | --- | --- |
| Ferritin  (ug/L) | 1 | 0.693 | 0.618 - 0.760 | <0.001 | 62.50% | 72.00% | 2758.5 |
|  | 2 | 0.729 | 0.638 - 0.808 | <0.001 | 88.60% | 61.40% | 1256 |
|  | 3 | 0.727 | 0.631 - 0.809 | <0.001 | 71.10% | 68.90% | 1543 |
|  | 4 | 0.760 | 0.668 - 0.837 | <0.001 | 65.70% | 79.20% | 1657.5 |
| ALT  (U/L) | 4 | 0.669 | 0.553-0.785 | 0.007 | 75.80% | 57.40% | 38.5 |
| WBC  (×10^9^/L) | 1 | 0.670 | 0.593-0.742 | <0.001 | 87.36% | 43.59% | 1.5 |
|  | 2 | 0.621 | 0.521-0.714 | 0.037 | 84.13% | 40.48% | 2.06 |
| NEUT (×10^9^/L) | 1 | 0.644 | 0.566-0.717 | 0.001 | 74.71% | 57.14% | 1.59 |
|  | 2 | 0.617 | 0.517-0.710 | 0.042 | 79.37% | 47.62% | 1.25 |
| HB  (g/L) | 1 | 0.642 | 0.563-0.715 | <0.001 | 47.13% | 76.92% | 88 |
|  | 2 | 0.742 | 0.647-0.822 | <0.001 | 73.02% | 69.05% | 83 |
|  | 4 | 0.68 | 0.578-0.771 | 0.002 | 56.92% | 78.79% | 97 |
| PLT  (×10^9^/L) | 1 | 0.764 | 0.692-0.826 | <0.001 | 82.76% | 61.54% | 57 |
|  | 2 | 0.740 | 0.645-0.821 | <0.001 | 55.56% | 83.33% | 128 |
|  | 3 | 0.766 | 0.671-0.845 | <0.001 | 70.18% | 76.74% | 127 |
|  | 4 | 0.724 | 0.624-0.809 | <0.001 | 89.23% | 57.58% | 72 |
| Ferritin/pla-  telet ratio | 1 | 0.769 | 0.697-0.831 | <0.001 | 69.20% | 74.70% | 32.32 |
|  | 2 | 0.776 | 0.685-0.852 | <0.001 | 78.60% | 69.80% | 16.08 |
|  | 3 | 0.800 | 0.709-0.874 | <0.001 | 60.50% | 91.20% | 30.70 |
|  | 4 | 0.772 | 0.677-0.851 | <0.001 | 66.70% | 81.50% | 21.97 |

Supplementary Table 5. Ferritin decline ratio for predicting induction response.

| Ferritin decline ratio | AUC | 95%CI | P-value | Sensitivity | Specificity | Cut-off value (lg/L) |
| --- | --- | --- | --- | --- | --- | --- |
| One week after treatment | 0.631 | 0.548-0.714 | 0.003 | 81.70% | 43.70% | 5.70% |
| Two weeks after treatment | 0.576 | 0.464-0.688 | 0.173 |  |  |  |
| Three weeks after treatment | 0.643 | 0.532-0.755 | 0.012 | 88.50% | 44.40% | -3.60% |
| Four weeks after treatment | 0.675 | 0.562-0.789 | 0.003 | 80.60% | 60.00% | 44.13% |

Supplementary Table 6. Comparison of the AUCs of serum ferritin, platelet, and the ferritin/platelet ratio.

| P-value | Ferritin vs. platelet | Ferritin vs. the ratio | Platelet vs. the ratio |
| --- | --- | --- | --- |
| One week after treatment | 0.077 | 0.002 | 0.827 |
| Two weeks after treatment | 0.689 | 0.082 | 0.296 |
| Three weeks after treatment | 0.629 | 0.025 | 0.422 |
| Four weeks after treatment | 0.457 | 0.716 | 0.127 |

Supplementary Figure 1 ROC analysis of serum ferritin decline ratio at 4 weeks after induction therapy in predicting induction response (AUC=0.675, 95% CI 0.562-0.789, P=0.003).


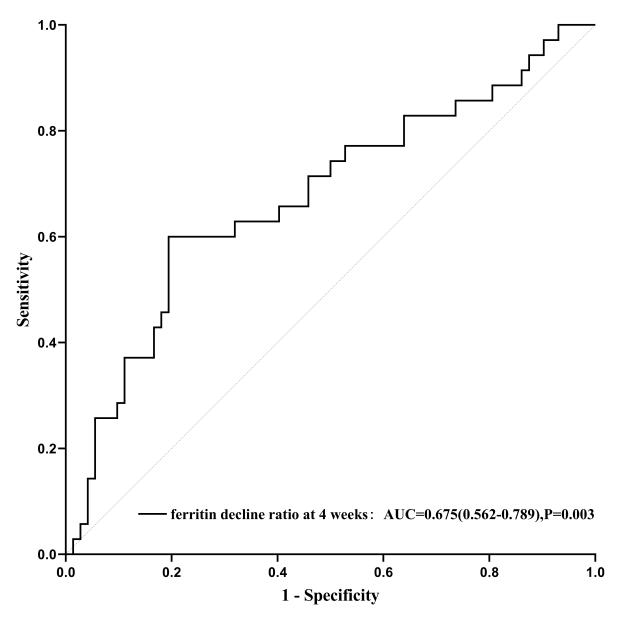


Supplementary Figure 2 Response to induction therapy in patients with different ALT stratification at 4 weeks after induction therapy was significant different. Four weeks: HR=0.317(0.143-0.704) P=0.005.
